# Total Demand, Use and Unmet Need for Modern Contraceptives Among Women Living in Informal Settlements in Kira Municipality, Wakiso District, Uganda. Implications for Urban Health

**DOI:** 10.3389/fgwh.2021.655413

**Published:** 2021-08-18

**Authors:** Moses Tetui, Mazen Baroudi, Tonny Ssekamatte, Catherine Birabwa, Simon Peter Kibira, Lynn Atuyambe, Alexandre Delamou, Fredrick Edward Makumbi

**Affiliations:** ^1^School of Pharmacy, Waterloo University, Waterloo, ON, Canada; ^2^Department of Epidemiology and Global Health, Umeå University, Umeå, Sweden; ^3^Department of Health Policy, Planning and Management, Makerere University School of Public Health, New Mulago Hospital Complex, Kampala, Uganda; ^4^Department of Disease Control and Environmental Health, Makerere University School of Public Health, New Mulago Hospital Complex, Kampala, Uganda; ^5^Department of Community Health and Behavioral Sciences, Makerere University School of Public Health, New Mulago Hospital Complex, Kampala, Uganda; ^6^Africa Center of Excellence for Prevention and Control of Communicable Diseases (CEA-PCMT), Faculty of Science and Health Techniques, Gamal Abdel Nasser University of Conakry, Conakry, Guinea; ^7^National Training and Research Center in Rural Health of Maferinyah, Forecariah, Guinea; ^8^Department of Epidemiology and Biostatistics, Makerere University School of Public Health, New Mulago Hospital Complex, Kampala, Uganda

**Keywords:** total demand, modern contraceptive use, unmet need, urban informal settlements, urban health, systems thinking

## Abstract

**Background:** Update and utilization of modern contraceptives has public health benefits including reduction of unintended pregnancies, unsafe abortions, and related maternal mortality. However, paucity of evidence on key indicators of family planning in the informal settlements abounds. Data are usually collapsed within the larger urban communities that tend to mask peculiarities of informal settlements. This study determined the proportion of women using modern contraceptives, the unmet need for modern contraceptives and the total demand in informal settlements of an urban municipality.

**Methods:** A cross-sectional study conducted among 626 women in the reproductive age (15–49 years) in the informal settlements of Kira municipality (part of metropolitan Kampala). Multi-stage sampling was applied in the selection of the respondents. Descriptive and log-binomial regression analysis were conducted to determine percentage of women using modern contraceptives, unmet need, and total demand with their associated factors. All analyses were conducted using STATA version 15.0.

**Results:** The total demand for modern contraceptives was 84.9%, modern contraceptive prevalence was 47.4% nearly meeting the national target of 50%, however the unmet need was 37.3%, which much higher than the national target of 10%. Lower total demand for contraceptives was associated with higher women's education status and preference to have another child, while higher total demand was associated with having at least one living child. Higher modern contraceptives use was associated with older age, having at least one living child and high decision-making power, while lower modern contraceptives use was associated with higher education and undetermined fertility preference. Lower unmet need for modern contraceptives was associated with older age (PR 0.68, 95% CI: 0.48–0.97) and high decision-making power (PR 0.64, 95% CI: 0.50–0.81), while higher unmet need was found among those who having at least one living child (PR 1.40, 95% CI: 1.01–1.93) and undetermined fertility preference (PR 1.70, 95% CI: 1.24–2.34).

**Conclusions:** Total demand and contraceptive use were found to be higher in the informal settlements of Kira municipality, however, the unmet need was much higher among this population as compared to the national urban estimates. This indicates a much higher demand for contraceptives and the need to consider the diverse socio-demographic characteristics of urban spaces. Development of Interventions need to critically consider the diverse urban space, associated explanatory variables and a collaborative systems lens to achieve sustained improvements.

## Background

Globally, women's access to family planning services remains a significant public health and social challenge. In 2019, only 842 million out of 1.11 billion women of reproductive age who needed a family planning service used a modern contraceptive method, while 270 million still had an unmet need (1). In addition, only 75.7% of the total demand for family planning was satisfied (1). The unmet need for family planning services is worse in the developing world where over 232 million women cannot access a modern contraceptive method when needed (2).

Given the public health and social benefits of family planning, several governments made commitments aimed at bridging access challenges. In 2012, for instance, Uganda committed to reduce the unmet need for family planning to 10%, and to increase the modern contraceptive prevalence rate to 50% by 2020 (3). The modern contraceptive rate has since 2001 increased from 22.8 to 39.0% in 2016, the total demand has increased from 54.0 (2001) to 67.0% (2019) while the unmet need reduced from 35.0 (2001) to 28.0% (2016) (4). An increase in the modern contraceptive prevalence rate and total demand consequently leads to improved maternal health outcomes.

Although there have been improvements in family planning indicators, a lack of disaggregated data on key family planning services within the urban settings could be masking the situation faced by the urban poor whose socio-economic status predisposes them to unique challenges (5). As a result, the urban population especially those living in informal settlements, defined as settings characterized by overcrowding, insecure land tenure, and limited access to social services, including healthcare services, remain under-served. Failure to improve family planning access rates for the urban poor compromises their ability to benefit from the demographic dividend (6). Indeed, a recent study that mapped family planning services in an urban setting in Uganda demonstrated a limited availability of family planning services in informal settlements as compared to formal settlements. For example, it was found that long-acting contraceptives were significantly less available in informal settlements, in addition, informal settlements were largely serviced by small private actors operating in one to two roomed facilities (7). This indicated limited access to choice and quality for persons living in informal settlements, factors that could potentially undermine the use of modern contraceptives.

Unplanned births constrain the already meager household finances and leads to poor child health outcomes such as malnutrition and unsafe abortions. Unsafe abortions are common due to Uganda's severely restricted abortion law, often resulting into maternal complications, disability, catastrophic expenditures and at times maternal deaths (8–11). Despite the consequences of unabated population growth and the imminent population sprawl that characterize urban areas especially informal settlements, there is limited evidence on the demand, unmet need and modern contraceptive prevalence among women living in informal settings. Existing literature often provides statistics along the dual urban and rural divide without examining the intra-urban differences. Usually, urban spaces are presented as having much more favorable indicators, thereby limiting the need to improve service investments in such settings (11, 12). For example, the latest UDHS indicated the unmet need among urban dwellers to be at 22.8% while that of their rural counterparts was 30.1% (4). Our study, therefore, determined the total demand, modern contraceptive prevalence and the unmet need for modern contraceptives, and associated factors among women living in informal settlements in Kira municipality, Uganda. Kira was selected given its representativeness of the rapid urban population growth that characterizes many medium sized urban centers in the country and the east Africa region. In addition, similar studies in the region have mainly focused on big cities with huge slums whose context could be different for a medium sized urban municipality such as Kira municipality (10, 13).

## Methods

### Study Design and Setting

This cross-sectional study was conducted in Kira municipality, Wakiso district Uganda. The study area serves as both the commercial and dormitory center for people working in Kampala, Uganda's Capital City. The municipality is located approximately 5.3 km by road, East of Kampala, and stands at latitude 0.397239 and longitude 32.640557 North of Equator. The estimated population of Kira municipality is over 400,000 inhabitants. It is made up of three divisions namely, Kira, Bweyogerere and Namugongo, with a total land area of about 98.83 km2 (4). The municipality is characterized by a high population sprawl, which has in-turn compromised physical planning and effective delivery of social services, including healthcare. The rapid population growth in Kira municipality has resulted in the growth of informal settlements. The informal settlements have developed mainly in the divisions of Bweyogerere and Namugongo, making these two divisions the specific study areas.

### Study Population and Eligibility

Our study population comprised of women in reproductive age (15–49 years old). To be a respondent, one must have been aged between 15 and 49 years at the time of the survey. All such women who were not residents and those who had not stayed in Kira Municipality for at least 6 months prior to the study were not eligible. Data were collected between 30 June and 10th July 2019.

### Sample Size Calculation

The sample size for this study was calculated using the Kish Leslie formula for cross-sectional studies (14). We assumed a prevalence of modern contraceptive use in an urban setting in Uganda of 52.1% (4), a 95% level of confidence, a margin of error (d) of 0.05 and a Z score of 1.96. This yielded a minimum sample size of 377. To adjust for non-response, we used the following formular.


$$\begin{array}{l} n=\frac{N}{1-x\;} \end{array}$$

Considering a non-response rate, x, of 10% and N, the estimated sample size, and substituting into the formula, a total sample size of 418 was established. In addition, a design effect of 1.5 was considered to adjust for the clustered nature of the sample across the two divisions that had slums in Kira Municipality. This brought the total sample size to 627 participants.

### Sampling

Multi-stage sampling was used in the selection of the respondents. The two study divisions were purposively selected since these comprised of the informal settings in the municipality. After the selection of the divisions, it was determined that a total of 8 villages were within the informal settlements of Kira municipality, four of these were randomly selected to be included in the study. A list of enumeration areas (EAs) used by Uganda bureau of statistics was then used to determine EAs that fall within these informal settlements, these totaled to 65. A random sample of 13 EAs was then selected. A listing of all households with eligible women followed in all the 13 EAs. Every EA has an average of 165 households, an equal sample size (49) was randomly selected from each of the EAs selected. The random sample was obtained using a mobile application named “RING Plus.” During interviewing, if an eligible woman was not available, three recalls were made before a replacement would be made, all eligible women were reached within the three recalls.

### Variable Measurement

#### Outcome Variables

- ***Unmet need***: The unmet need was defined based on the methods developed by Bradley et al. (15). Unmet needs were coded as 1 if a married/in union or sexually active unmarried woman is fecund, not using a modern contraceptive method AND

∘ Are not pregnant or postpartum amenorrhoeic OR
∘ Their current pregnancy/last birth is/was unwanted or wanted later OR
∘ Does not want to have more children or wants to delay the next child by 2+ years or undecided
∘ Sexually active women are those who had sex during the last month.

- ***Modern contraceptive use***: women who are currently using any modern (male or female sterilization, IUD, implant, injectables, pills, emergency pills, male and female condoms, diaphragm, and foam jelly) contraceptive method were coded as 1.
- ***Total demand***: was coded as 1 if the woman is currently using any modern contraceptive method or have unmet needs.

#### Covariates

The following covariates were selected to be used in this study based on their use in existing literature, the Uganda demographic health survey and in the performance monitoring for action (PMA 2020) project (4, 9, 10, 16–19).

- ***Age***: was categorized as 15–24 years-old, 25–34 years-old and 35–49 years old.
- ***Number of living children*** was coded as 1 if the women have at least one living child.
- ***Women' education*** were categorized as (1) no education if the woman had never attended school or never completed primary school, (2) primary if the women completed primary school and (3) secondary and higher if the women have attended ordinary secondary school (4 years post primary), Advanced secondary school (6 years post primary) or tertiary/University.
- ***Marital status*** was categorized as (1) unmarried or not in union including widows and divorced women and (2) married or in union.
- ***Experienced death of a child*** was coded as 1 if the women have ever experienced a death of a child regardless of the child's sex.
- ***Fertility preference*** was categorized as (1) no more children desired and (2) want another child or (3) undecided.
- ***FP counseling*** was coded as 1 if during the last 12 months the women visited a FP facility or been visited by a community health worker who talked about FP.
- ***Woman's decision-making power*** was coded as empowered if the women (1) can say no to having sex, (2) can avoid having sex if not wanted, (3) if she wanted to use contraceptives she can tell her partner, and (4) can use contraceptives whenever she wants. The women were coded as “less empowered” if they cannot do at least one of these four mentioned criteria.
- ***Media exposure***: was coded as 1 if the women have received information about FP through radio, TV, or newspaper/magazine.

### Data Collection Tool and Data Management Plan

Data were collected using a women's questionnaire developed under the performance monitoring for action (PMA 2020) project. The tool is used to collect data on family planning indicators in elven countries (19). The tool is consistent with DHS tools, which have already been validated. The key areas included socio-demographics, reproduction, pregnancy and fertility preferences, contraception, sexual activity, and empowerment. To allow for real time data capture and entry, and to minimize errors and missing data during field data collection, the questionnaire was preloaded on the Kobo Collect mobile application. The e-questionnaire was designed with relevant skips to allow for consistency during data collection. The research assistants uploaded data collected to the server daily. Upon submission of the data to the server, the investigators and data managers conducted quality control checks on key variables such as age to ascertain their correctness.

### Statistical Analysis

The outcome variables: total demand, modern contraceptives use and unmet need, were calculated and prevalence presented by the respondents' characteristics. The associations between the three outcomes and the respondents' characteristics were examined using log- binomial regression (20). Unadjusted prevalence ratios with their 95% confidence intervals were calculated and presented for each of the outcomes. The adjusted prevalence ratios were calculated after controlling for all explanatory variables. Regression models were tested for multicollinearity with the variance inflation factor. A log-binomial regression was used since our outcomes were estimated to be more than 10% given existing studies. In such cases, Odd ratios derived from logistic regression would overstate values compared to prevalence ratios derived from log-binomial regression (21). Sexually inactive unmarried women and infecund women^1^ were not included in the data analysis, this brought the overall total analyzed sample size to 477 women of reproductive age.

### Quality Assurance and Quality Control

Research assistants were trained on the research protocol and ethical issues surrounding the study to ensure quality data collection. TM and TS, together with the RAs conducted a pretest of the data collection tools. The pretest was conducted in Katanga, a slum in Kampala, Uganda's capital. Katanga was selected as the ideal setting for the pre-test since it had similar characteristics to those of the selected enumeration areas in Kira municipality. Just like the selected enumeration areas, Katanga slum is heavily populated, limited or no physical planning and limited social service provision.

## Results

### Socio-Demographic Characteristics of the Respondents

A total of 626 women of reproductive age were interviewed, representing a response rate of 99.8%. However, to undertake the analysis, sexually inactive unmarried women and infecund women where dropped, making the sample size 477. The average age of the participants was 28.3 (standard deviation = 7.5). About 47.6% (227/477) of the participants were aged between 25 and 34 years, 82.4% had at least one living child and 38.5% (183/477) had attained primary education as their highest level. Around one tenth of the respondents, 11.1% (53/477) had ever experienced death of their child. Majority, 52.2% (249/477) were empowered and 71.9% (343/477) had mainstream (e.g., radio, TV, or newspaper) media exposure to family planning information (Table 1).

**Table 1:** Background characteristics of the study participants.

**Variable**	**Frequency (*N* = 477)**	**Percentage (%)**	**95% confidence interval**
**Age (Years)**			
15–24	166	34.8	30.6–39.2
25–34	227	47.6	43.1–52.1
35–49	84	17.6	14.4–21.3
**Woman's education**			
No education	27	5.7	3.9–8.2
Primary	183	38.5	34.2–43.0
Secondary & higher	265	55.8	51.3–60.2
**Marital status**			
Unmarried/not in union	47	10.0	7.6–13.0
Married/in union	425	90.0	87.0–92.4
**Number of living children**			
No living children	84	17.6	14.4–21.3
At least 1	393	82.4	78.7–85.6
**Fertility preference**			
No more children desired	96	20.4	17.0–24.3
Another child	309	65.7	61.3–69.9
Undecided	65	13.8	11.0–17.3
**Experienced death of a child**			
Yes	53	11.1	8.6–14.3
No	424	88.9	85.7–91.4
**Family planning counseling**			
Yes	271	56.8	52.3–61.2
No	206	43.2	38.8–47.7
**Woman's decision-making power**			
Less empowered	228	47.8	43.3–52.3
Empowered	249	52.2	47.7–56.7
**Media exposure**			
Yes	343	71.9	67.7–32.3
No	134	28.1	24.2–32.3

### Total Demand, Modern Contraceptive Use, and Unmet Need

Overall, 84.9% (405/477) of the participants had demand for a modern contraceptive method. The prevalence of modern contraceptive use was 47.4% (226/477), and the unmet need was 37.3% (178/477). Based on the socio-demographic strata, the total demand for contraceptives was highest among women who had no formal education-100% (27/27) but they had 37% (10/27) unmet need. In terms of modern contraceptive use, this was highest among women who had no formal education, had no desire for more children (59.4%), and were empowered (59.0%).

The unmet need for family planning was highest among those who were undecided about getting another child-64.6% (42/65), those who were less empowered women-46.9% (107/228) and those who had experienced death of a child-43.4% (23/53) (Table 2).

**Table 2:** Prevalence of total demand, modern contraceptive use and unmet needs.

**Variable**	**Total demand *n* (%)**	**Modern contraceptive use *n* (%)**	**Unmet** **need *n* (%)**
Overall^*^	405 (84.9)	226 (47.4)	178 (37.3)
**Age category (Years)**			
15–24	128 (77.1)	61 (36.8)	67 (40.4)
25–34	203 (89.4)	125 (55.1)	77 (33.9)
35–49	74 (88.1)	40 (47.6)	34 (40.5)
**Woman's education**			
No education	27 (100.0)	17 (63.0)	10 (37.0)
Primary	154 (84.2)	87 (47.5)	67 (36.6)
Secondary & higher	223 (84.2)	122 (46.0)	100 (37.7)
**Marital status**			
Unmarried/not in union	38 (80.9)	19 (40.4)	19 (40.4)
Married/in union	365 (85.9)	207 (48.7)	157 (36.9)
**Number of living children**			
No living children	48 (57.1)	19 (22.6)	28 (33.3)
At least 1	357 (90.8)	207 (52.7)	150 (38.2)
**Fertility preference**			
No more children desired	93 (96.9)	57 (59.4)	36 (37.5)
Another child	254 (82.2)	153 (49.5)	100 (32.4)
Undecided	57 (87.7)	15 (23.1)	42 (64.6)
**Experienced death of a child**			
Yes	47 (88.7)	24 (45.3)	23 (43.4)
No	358 (84.4)	202 (47.6)	155 (36.6)
**Family planning counseling**			
Yes	236 (87.1)	133 (49.1)	103 (38.0)
No	169 (82.0)	93 (45.2)	75 (36.4)
**Woman's decision-making power**			
Less empowered	186 (81.6)	79 (34.7)	107 (46.9)
Empowered	219 (87.9)	147 (59.0)	71 (28.5)
**Media exposure**			
Yes	106 (79.1)	56 (41.8)	50 (37.3)
No	299 (87.2)	170 (49.6)	128 (37.3)

* *the percentages in this row is the percentage from the total sample*.

Total demand for contraceptives was associated with women's education; women with secondary or higher education had lower demand compared to those with no education (PR 0.87, 95% CI: 0.80–0.94). Those having at least one living child had higher demand compared to those having no living child (PR 1.49, 95% CI: 1.24–1.79) and those preferring to have another child had lower demand compared to those who preferred having no more child (PR 0.90, 95% CI: 0.84–0.97).

Regarding modern contraceptive use, women aged 25–34 years had higher use compared to younger women (PR 1.35, 95% CI: 1.07–1.69), women with secondary or higher education status had lower use compared to women with no education (PR 0.73, 95% CI: 0.53–0.99), women who had at least one living child had higher use compared to those who had none (PR 1.74, 95% CI: 1.15–2.63), women who were uncertain about their fertility preference had lower use compare to those who preferred not having more children (PR 0.48, 95% CI: 0.30–0.77) and empowered women had higher use compared to the less empowered (PR 1.45, 95% CI: 1.18–1.78).

The unmet need for modern contraceptives was associated with age; older women had lower unmet need compared to younger women (PR 0.68, 95% CI: 0.48–0.97), women with at least one living child had higher unmet need compared to those who had none (PR 1.40, 95% CI: 1.01–1.93), women who were uncertain about their fertility preference had higher unmet need compared to those who preferred not having more children (PR 1.70, 95% CI: 1.24–2.34) and empowered women had lower unmet need compared to the less empowered (PR 0.64, 95% CI: 0.50–0.81 (Table 3).

**Table 3:** Factors associated with total demand, modern contraceptive use and unmet need for modern contraceptives among women of reproductive age in Kira Municipality, Uganda.

**Variable**	**Total demand**		**Modern contraceptive use**		**Unmet need**	
	**Crude PR^***a***^**	**Adjusted PR^***b***^**	**Crude PR**	**Adjusted PR**	**Crude PR**	**Adjusted PR**
**Age category (Years)**						
15–24	1	1	1	1	1	1
25–34	1.16^*^ (1.06, 1.27)	1.02 (0.93, 1.11)	1.50^*^ (1.19, 1.89)	1.35^*^ (1.07, 1.69)	0.84 (0.65, 1.09)	0.68^*^ (0.52, 0.88)
35–49	1.14^*^ (1.02, 1.28)	1.00 (0.89, 1.13)	1.30 (0.96, 1.75)	1.33 (0.98, 1.81)	1.00 (0.73, 1.38)	0.68^*^ (0.48, 0.97)
**Woman's education**						
No education	1	1	1	1	1	1
Primary	0.84^*^ (0.79, 0.90)	0.85^*^ (0.78, 0.92)	0.76 (0.54, 1.05)	0.74 (0.54, 1.02)	0.99 (0.58, 1.68)	1.07 (0.67, 1.69)
Secondary & higher	0.84^*^ (0.80, 0.89)	0.87^*^ (0.80, 0.94)	0.73 (0.53, 1.00)	0.73^*^ (0.53, 0.99)	1.02 (0.61, 1.71)	1.11 (0.71, 1.75)
**Marital status**						
Unmarried/not in union	1	1	1	1	1	1
Married/in union	1.06 (0.92, 1.23)	0.89 (0.78, 1.02)	1.20 (0.84, 1.73)	0.86 (0.60, 1.24)	0.91 (0.63, 1.32)	0.92 (0.63, 1.34)
**Number of living children**						
No living children	1	1	1	1	1	1
At least 1	1.59^*^ (1.32, 1.92)	1.49^*^ (1.24, 1.79)	2.33^*^ (1.55, 3.50)	1.74^*^ (1.15, 2.63)	1.15 (0.83, 1.59)	1.40^*^ (1.01, 1.93)
**Fertility preference**						
No more children desired	1	1	1	1	1	1
Another child	0.85^*^ (0.80, 0.90)	0.90^*^ (0.84, 0.97)	0.83 (0.68, 1.02)	0.96 (0.78, 1.18)	0.86 (0.64, 1.17)	0.83 (0.61, 1.13)
Undecided	0.91^*^ (0.82, 1.00)	0.98 (0.87, 1.09)	0.39^*^ (0.24, 0.62)	0.48^*^ (0.30, 0.77)	1.72^*^ (1.26, 2.36)	1.70^*^ (1.24, 2.34)
**Experienced death of a child**						
No	1	1	1	1	1	1
Yes	1.05 (0.95, 1.17)	0.98 (0.88, 1.08)	0.95 (0.70, 1.30)	0.90 (0.66, 1.23)	1.19 (0.85, 1.65)	1.09 (0.78, 1.52)
**FP counseling**						
No	1	1	1	1	1	1
Yes	1.06 (0.98, 1.15)	1.02 (0.95, 1.11)	1.09 (0.90, 1.32)	1.09 (0.90, 1.33)	1.04 (0.82, 1.32)	0.94 (0.73, 1.22)
**Woman's decision-making power**						
Less empowered	1	1	1	1	1	1
Empowered	1.08 (1.00, 1.16)	1.03 (0.95, 1.11)	1.70^*^ (1.39, 2.09)	1.45^*^ (1.18, 1.78)	0.61^*^ (0.48, 0.77)	0.64^*^ (0.50, 0.81)
**Media exposure**						
No	1	1	1	1	1	1
Yes	1.10^*^ (1.00, 1.21)	1.07 (0.98, 1.17)	1.19 (0.95, 1.49)	1.11 (0.89, 1.38)	1.00 (0.77, 1.30)	1.01 (0.78, 1.32)

* *P-value < 0.05.*

a *Crude PR, Unadjusted prevalence ratios (the result of the uni-variable regressions).*

b *Adjusted PR, The adjusted prevalence ratios were calculated after controlling for all explanatory variables*.

## Discussion

Our study aimed at establishing the total demand, prevalence of modern contraceptive use, and the unmet need for modern contraceptives among women living in informal settlements in a highly populated urban setting in Uganda. The total demand for contraceptives was 84.9%, less than half (47.4%) of the women in the study area were current users of a modern contraceptive method, and at least 37.3% had an unmet need for family planning. Modern contraceptives are highly efficacious in reducing unplanned, unwanted, and mistimed pregnancies. Closing the demand-use gap is therefore essential.

The unmet need for modern contraceptives in informal settlements for married (36.9%) and unmarried sexually active women (40.4%) was found to be considerably higher than the urban estimates (22.8%) and (22.2%), respectively, as reported in the most recent demographic health survey (4). A fact that is often masked by the national demographic surveys that report findings of urban spaces as though the urban space is demographically homogenous and usually with better outcomes as compared to rural areas (11, 12, 22). In Uganda, rapid growth of the urban population has not grown at the same pace as the needed physical infrastructure to meet social needs such as education, housing, and health care for all. This, consequently, has led to the creation of informal settlements that are often underserved by social services (12). In addition, the unmet need for family planning remains much higher than the national target of 10% (23). The unmet need for family planning in informal settings could be attributed to inadequate availability of service delivery points, limited information on family planning services, and the low socio-economic status that deprives the population of the ability to pay for family planning services when needed (7, 16). Interestingly, the total demand and modern contraceptive use in this population was much higher than what is reported for the general urban population (4). Similarly, this could be attributed to the lower socio-economic status of this population, social context within which women live has been shown to influence contraceptive use (7, 16, 24).

Total demand in this study was inversely associated to education level of women, a finding that we found surprising. Further studies within similar population should be conducted to validate this. Nonetheless, despite the high total demand and use among women with no education, the unmet need is still comparable to those with higher education. The role of education in empowering women to take more charge of their sexual and reproductive health issues is indisputable (4, 17, 25). Indeed, our study still found a high total demand (84.2%) and contraceptive use (46.0%) among the highly educated women in the informal settlements of Kira municipality. Moreover, a study conducted in Uganda found a link between education and increased contraceptive use by increasing their economic opportunities and likelihood of engaging in protected sex (18).

Total demand and modern contraceptive use among women living in informal settings was higher if the woman had at least one living child, still unmet need is higher in this group. The use of modern contraceptives in many African settings is often dictated by the number of children a woman bears. Women who fail to bear children are often disregarded in society (26, 27). Therefore, women without any children tend to have a lower modern contraceptive demand as a way of increasing their chances of bearing children for their partners. This implies that after bearing children, women are in a better position to decide whether to have more children or not, and thus an increase in the total demand and modern contraceptive use. Findings indicating the role of having at least one living child in shaping modern contraceptive prevalence have also been reported other parts of Sub-Saharan Africa (27, 28).

Similarly, fertility preference was associated with total demand for modern contraceptives, use, and unmet need. Women who were undecided about having another child had a much higher unmet need (64.6%). Such women are at a higher risk of unintended or mistimed pregnancies compared to their counterparts who wanted no more children. Being undecided places women in an uncertain circumstance which increases their chances of unintended pregnancies. In Uganda, it is often the decision of the men to decide how many children to have and when to (29). This could be compounded by limited access to family planning services such as health education and counseling in informal settings (7, 26). The need for a broader approach to sexual and reproductive health issues, to include fertility preferences and the role of women is essential. Additionally, this highlights the wider cultural context within which contraceptive use choices are made and underscores the need for a multi-sectoral/systems approach to improving the use of modern contraceptives (28, 30). For example, as expounded in the next paragraph, more empowered women are more likely to be in charge of their fertility choices.

Modern contraceptive use was higher among the empowered women in comparison to the less empowered and therefore had lower unmet need. Empowerment improves the agency of women thus making it easier for them to make decisions on issues pertaining their sexual and reproductive health. Unlike less empowered women, empowered women are often in a better position not to have sex when they want, and to use contraceptives whenever they want. The impact of women's empowerment on modern contraceptive use is widely documented in other settings (31, 32), and this indeed underscored the need to empower women in order for them to realize their sexual and reproductive health needs. These findings indicate the critical role that gender equality interventions could play in advancing the use of modern contraceptives (32–34). This, therefore, calls for a more multifaceted approach to promoting contraceptive use, thereby making a systems thinking lens critical in intervention designs.

Further, the prevalence of modern contraceptive use was lowest among young people (15–24 years). Modern contraceptive use was lowest among the 15–24-year-olds probably due to the healthcare system barriers that characterize informal settings. The age group represents a cluster of young adults whose sexual and reproductive health care needs are often unique, thereby in need of youth focused services. Service delivery points/healthcare facilities in such settings often lack access to youth-friendly sexual and reproductive health services (7). Yet, such services are known to improve the health seeking behaviors of young people (35). Absence of youth-friendly services limits the uptake of modern contraceptives since young people may shy away from using service delivery points that are dominated by the older age groups due to fear of being judged, and at times lack of the financial power to pay for family planning services. The lack of youth-friendly services also limits the sensitization of young people on the use of modern contraceptives, as well as contraceptive counseling, both of which are known to increase uptake of modern contraceptives and consequently reduce the unmet need for modern contraceptives (7, 36). The current study highlights the need for innovative solutions aimed at increasing access to modern contraceptives to young people living in informal settlements.

### Methodological Considerations

The current study offers a unique dataset to understand modern contraceptive use and unmet need in informal settlements. It follows a rigorous sampling and data collection method with a very high response rate to obtain a representative sample of women of reproductive age in a mid-sized informal settlement. However, several points should be considered when interpreting our findings. First, to undertake the study analysis, we excluded infecund and sexually inactive unmarried women in keeping with the revised definition of unmet need. This, however, appears to have led to higher estimates compared to the Uganda demographic health survey (UDHS) which does not exclude the infecund and sexually inactive from the analysis undertaken. Our findings, therefore, should be interpreted considering the differences in sample characteristics.

Second, given the cross-sectional design of the study, all results should be understood as associations rather than causal relations. Third, we combined single women and those divorced or widowed during data analysis. We believe this did not have had any effect on the results of the study. However, we recommend that these be separated for future studies to understand the differences within these groups as pertains the outcomes of interest. Finally, we think that our study area is a good example of mid-size urban areas in East Africa and therefore our results can be generalized to other areas with similar settings.

## Conclusions and Recommendations

The total demand and modern contraceptive use in this study were found to be high. The prevalence of modern contraceptive use was lowest among women aged 15–24.

The unmet need in this study (37.3%) was found to be much higher than what is reported in the latest UDHS (22.8%) for the urban population. This is an indication of the socio-demographic and socio-economic diversity of the urban population. Unmet need for modern contraceptive use was associated with women having at least one living child, their fertility preferences and empowerment levels.

To decrease unmet need for contraceptive use among residents of urban informal settlements, we make three interrelated recommendations. One, interventions targeted for the urban population need to take into consideration the diverse socio-demographic and social economic characteristics of the urban population. Those living in informal settlements are faced with unique challenges to contraceptive access and use, understanding those and tailoring interventions to overcome them is critical. A further look into the drivers of high demand and use of modern contraceptives among women living in informal settlements is recommended. We hypothesize that the high urban living costs, could be a motivation to limit childbearing amongst this population.

Two, the role of education, fertility preferences, having a living child and women's empowerment in determining contraceptive use among women living in formal settlements must be considered in intervention designs. Three, a whole systems approach to promoting contraceptive use in urban informal settlements will have higher chances of yielding positive results. Women's empowerment through promotion of quality girl child education, the use of micro-economic tools such as provision of soft loans to improve women's and youth (15–24-year-olds) income levels, promotion of school health programs and the engagement of men and other social actors to systematically reflect and change suppressive social and gender norms must be tackled from a multi-sectoral and systems perspective. Such a perspective will enable the harnessing of strengths from different stakeholders while at the same time enabling a holistic approach to needed changes, thereby creating opportunities for dealing with unintended consequences that may be created by making one off and stand-alone interventions. For example, to tackle entrenched gendered norms or limited use of contraceptives among the youth, will require concerted efforts of religious and other opinion leaders, politicians and cultural heads, local interest groups, youth leaders, civil society, men, education stakeholders, health workers, urban planners, and development partners. Such a complex approach needs to be sustained over a long period of time for it to yield intended benefits.

## Data Availability Statement

The raw data supporting the conclusions of this article will be made available by the authors, without undue reservation.

## Ethics Statement

Ethical approval was obtained from Makerere university School of Public Health Higher Degrees and Research Ethics Committee (HDREC-684). The study was registered with the Uganda National Council of Science and Technology (HS382ES). Permission to interview the participants was sought from Wakiso District local government and the Kira Municipality officials. Prior to any interviews, informed written consent was sought from all adults above 18 years, the legal age in Uganda. For minors (15–17 years), informed consent was first obtained from their parents/guardians, as well as assent from the respondents. Minors who were pregnant or those who had given birth at the time of the interviews were considered emancipated and thus consented on their own.

## Author Contributions

MT conceptualized the study, participated in data collection and management, and led the manuscript drafting. MB led data analysis with support from CB, MT, and SK. TS participated in data collection and supported the drafting of the manuscript. LA contributed to the conceptualization of the study. FM provided overall technical guidance to the conceptualization process and participated in reviewing initial drafts of manuscript. All authors reviewed the manuscript, provided substantial input, and approved the final manuscript.

## Conflict of Interest

The authors declare that the research was conducted in the absence of any commercial or financial relationships that could be construed as a potential conflict of interest.

## Publisher's Note

All claims expressed in this article are solely those of the authors and do not necessarily represent those of their affiliated organizations, or those of the publisher, the editors and the reviewers. Any product that may be evaluated in this article, or claim that may be made by its manufacturer, is not guaranteed or endorsed by the publisher.

## Footnotes

^1^The following conditions were identified as infecund: (1) not having a child in the last five years despite being married and never used contraceptives, (2) not having a period in the last 6 months and not postpartum amenorrhic and (3) those who answered “cannot get pregnant” when asked about their fertility preference.
